# Insights into Asparaginase Allergic Responses: Exploring Pharmacogenetic Influences

**DOI:** 10.3390/pharmaceutics16091134

**Published:** 2024-08-28

**Authors:** Daiane Keller Cecconello, Klerize Anecely de Souza Silva, Evelin Cristine Mendonça de Senna, Ciliana Rechenmacher, Liane Esteves Daudt, Mariana Bohns Michalowski

**Affiliations:** 1 Post Graduate Program in Child and Adolescent Health, Universidade Federal do Rio Grande do Sul, Porto Alegre 90035-003, RS, Brazil; daia_cecconello@hotmail.com (D.K.C.); klerize.anecely@gmail.com (K.A.d.S.S.); cilianare@gmail.com (C.R.); ldaudt@hcpa.edu.br (L.E.D.); 2 Translational Pediatrics Laboratory, Experimental Research Center, Hospital de Clínicas de Porto Alegre, Porto Alegre 90035-903, RS, Brazil; 3 Postgraduate in Pediatrics, Universidade de São Paulo, São Paulo 05508-220, SP, Brazil; evelinsenna3@gmail.com

**Keywords:** asparaginase, pharmacogenetics, hypersensitivity, acute lymphoblastic leukemia, single-nucleotide variant

## Abstract

Acute lymphoblastic leukemia represents the most prevalent childhood cancer. Modern chemotherapy has significantly improved outcomes, achieving EFS rates of 80% and OS rates nearing 90% in developed nations, while in developing regions, rates remain below 50%, highlighting disparities, and this difference is due to several factors. Genetic variability plays a role in these drug response disparities, presenting single-nucleotide variations (SNVs). Pharmacogenetic research aims to pinpoint these SNVs early in treatment to predict specific drug responses effectively. This review aims to explore advancements in pharmacogenetics associated with asparaginase (ASNase). ASNase plays a crucial role in the treatment of ALL and is available in three formulations: *E. coli*, *Erwinia*, and PEG ASNase. ASNase therapy presents challenges due to adverse effects, like hypersensitivity reactions. Identifying predictive markers for hypersensitivity development beforehand is crucial for optimizing treatments. Several pharmacogenetic studies have investigated the association between SNVs and the risk of hypersensitivity. Key genes include *GRIA1*, *NFATC2*, *CNTO3*, *ARHGAP28*, *MYBBP1A*, and *HLA*. Studies have highlighted associations between SNVs within these genes and hypersensitivity reactions. Notably, most pharmacogenetic investigations of hypersensitivity have focused on patients treated with *E. coli*, emphasizing the need for broader exploration across different formulations. Future research investigating these variants holds promise for advancing our understanding of ASNase’s pharmacogenetics.

## 1. Introduction

Acute lymphoblastic leukemia (ALL) represents the most prevalent childhood cancer, comprising approximately 75–80% of pediatric leukemia cases [1]. Modern chemotherapy protocols have significantly improved long-term outcomes, achieving event-free survival (EFS) rates of 80% and overall survival (OS) rates nearing 90% in developed nations. However, cure rates remain below 50% in developing regions, highlighting critical disparities that necessitate thorough investigation into regional complexities. This difference is due to several factors, including low socioeconomic status, scarcity of some antineoplastic agents, and lower investment in supportive care. Understanding why certain patients respond differently to established treatments remains a crucial pursuit [2,3].

Patient-specific factors, such as sex, age, and ethnicity, contribute to variations in drug responses across different treatments [4,5]. Genetic variability, manifested through over 640 million single-nucleotide variations (SNVs) in the human genome, plays a pivotal role in these drug response disparities [6]. These genetic polymorphisms, arising from chance or external factors, significantly influence metabolic pathways, thereby impacting treatment effectiveness. Identifying these polymorphisms is crucial for understanding why treatments succeed in some individuals while failing in others and for explaining variations in blood concentrations leading to therapeutic or toxic effects [7,8]. Pharmacogenetic research aims to pinpoint these genetic variants early in treatment to predict specific drug responses effectively [8,9].

In ALL, pharmacogenetic studies hold significant promise for several reasons. Firstly, chemotherapy drugs used in ALL treatment often have a narrow therapeutic range, where the difference between an effective dose and a toxic one is minimal. Moreover, genetic variations in the metabolic pathways of these drugs contribute to variability in drug exposure and activity, profoundly influencing treatment responses [10].

Given these complexities, there is a growing consensus among researchers regarding the critical role of genetic profiling in tailoring personalized therapy. This approach aims to maximize treatment efficacy while minimizing drug-related safety concerns [6,8,10]. By identifying specific genetic traits, researchers can elucidate (1) the genes encoding crucial proteins that influence drug responses; (2) variants within these genes, known as polymorphisms, that alter enzymatic activity or drug-binding capabilities; (3) scenarios where the same protein/enzyme affects drug metabolism differently; and (4) other influential factors impacting drug metabolism [11,12].

This review aims to explore recent advancements in pharmacogenetic variations associated with asparaginase, providing context for their future clinical applications.

## 2. Asparaginase (ASNase) in ALL Treatment

Asparaginase (ASNase) plays a crucial role in the treatment of acute lymphoblastic leukemia (ALL), typically administered during the induction and consolidation therapy phases [13]. It operates by depleting the non-essential amino acid asparagine (Asn) systemically, inducing apoptosis in neoplastic lymphoblastic cells characterized by low levels of asparagine synthetase. This targeted approach involves converting circulating Asn into aspartic acid and ammonia extracellularly, thereby reducing intracellular Asn levels and hindering protein synthesis, selectively affecting neoplastic cells while sparing normal cells capable of synthesizing Asn intracellularly [14,15].

ASNase is available in three formulations: derived from *Escherichia coli* or *Erwinia carotovora* (*Erwinia chrysanthemia*), and PEG ASNase resulting from the conjugation of *E. coli* ASNase with polyethylene glycol (PEG). PEG ASNase aims to minimize immunogenicity, decrease infusion frequency, and prolong serum half-life. Notably, PEG-ASNase exhibits an extended half-life (~7 days) compared with *E. coli* ASNase (1.3 days) and Erwinia ASNase (0.65 days), primarily due to reduced antibody formation and allergic responses [16,17,18,19]. The pharmacokinetic data on the formulations are shown in Table 1.

Despite its critical role, ASNase therapy presents substantial challenges due to adverse effects, significantly impacting patient morbidity and mortality [12,20]. Clinical hypersensitivity is a primary concern, often necessitating therapy discontinuation. Symptoms range from mild manifestations, such as injection site pain and urticaria, to severe reactions, including bronchospasm and hypotension. Notably, antibodies can target not only the protein but also the polyethylene glycol moiety, complicating treatment efficacy [21,22,23].

Hypersensitivity rates vary among ASNase formulations, at 10–75% for *E. coli* ASNase, 3–24% for PEG-ASNase, and 3–37% for Erwinia ASNase [21]. Hypersensitivity reactions may occur after initial exposure but are more common with repeated administrations during the consolidation and reinduction phases. This immune response involves the production of anti-ASNase antibodies that can reduce ASNase’s effectiveness [22]. Some patients experience reduced ASNase activity due to neutralizing antibodies without clinical symptoms, known as “silent inactivation”, affecting treatment efficacy [23,24,25]. ASNase hypersensitivity encompasses clinical allergies from mild rash to anaphylaxis, and silent inactivation denotes activity reduction without symptoms, both necessitating a switch to another ASNase formulation. Additional allergy-like reactions mimic hypersensitivity symptoms but do not affect ASNase activity, allowing treatment continuation if symptoms are mild [26].

**Figure 1:**
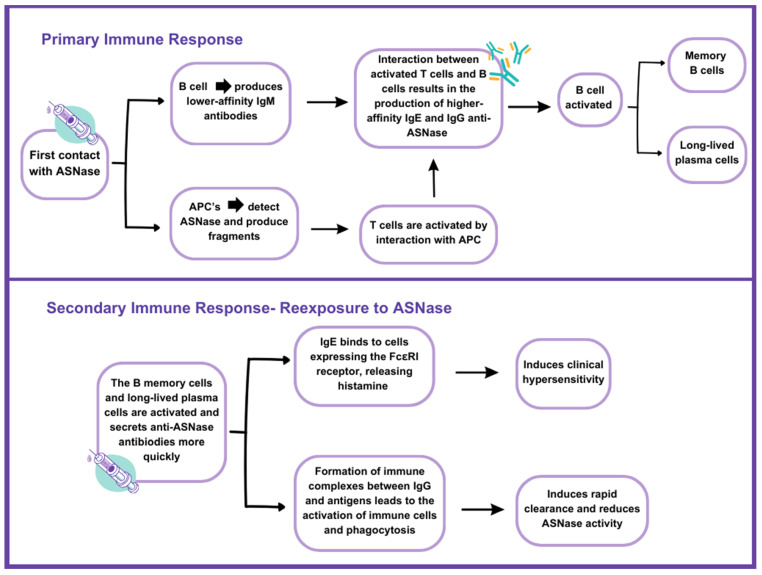
Mechanism of immune response to ASNase upon initial and subsequent exposures. Adapted by Burke et al. (2022) [22].

Upon ASNase exposure, initial immune responses involve circulating B cells producing low-affinity anti-ASNase immunoglobulin M (IgM) antibodies. Concurrently, antigen-presenting cells (APCs) process and present ASNase peptide fragments to T helper (Th) cells via major histocompatibility complex molecules. Activated Th cells then interact with antigen-specific B cells, prompting a class switch from IgM to higher-affinity immunoglobulin E (IgE) and immunoglobulin G (IgG) anti-ASNase antibodies. Memory B cells and long-lived plasma cells are subsequently activated, facilitating faster antibody secretion upon re-exposure [22,27]. IgE antibodies bind FcεRI receptors, triggering histamine release and hypersensitivity symptoms upon antigen binding. IgG antibodies form immune complexes with ASNase, binding Fcγ receptors and inducing phagocytosis, contributing to ASNase clearance and reduced activity. Elevated IgG levels in hypersensitive patients correlate with ASNase inactivation, suggesting potential early detection markers for clearance changes (Figure 1) [22,26].

Measures such as steroid premedication, which stabilizes mast cells and prevents the release of certain pro-inflammatory mediators, and desensitization techniques can mitigate symptoms but do not prevent the inactivation of ASNase activity due to the development of anti-ASNase antibodies, which can compromise treatment efficacy. Therapeutic drug monitoring (TDM) using serum ASNase activity (SAA) measurements is, therefore, crucial in current clinical guidelines for ASNase-based regimens. A trough SAA level of ≥0.1 IU/mL is generally considered necessary for achieving complete serum asparagine depletion. Once hypersensitivity occurs, continuing with the same ASNase formulation is not recommended. Switching to an ASNase variant with a different immunogenic profile allows more than 90% of patients to successfully complete their prescribed regimen [28,29,30].

## 3. Genetic Polymorphisms

Each individual’s response to treatment significantly varies, underscoring the importance of assessing clinical tolerance to balance drug toxicity with treatment efficacy in ALL patients. While escalating ASNase doses has shown potential to improve response rates, hypersensitivity reactions remain a common challenge, often leading to treatment discontinuation. Therefore, identifying predictive markers for hypersensitivity development beforehand is crucial for optimizing treatment strategies [10,20].

Several pharmacogenetic studies have investigated the association between gene polymorphisms and the risk of ASNase hypersensitivity. Key genes frequently studied include GRIA1, NFATC2, CNTO3, ARHGAP28, MYBBP1A, and HLA [10,31,32,33].

### 3.1. GRIA1

The GRIA1 gene encodes one subunit (GluR1) of the four (GluR1–4) of the AMPA (α-amino-3-hydroxyl-5-methyl-4-isoxazole-propionate) receptor, a tetrameric ligand-gated ion channel that transmits glutamatergic signals in the brain, forming Ca^2+^-permeable ion channels and mediating a fast excitatory glutamate response. It is located on chromosome 5q31-33, which contains a cluster of cytokine and other immune-related genes, such as interleukin-4, interleukin-13, and interleukin-5. This region has been mapped as a susceptibility locus for several immune and anti-inflammatory diseases and has been associated with asthma and atopy [31,34,35]. GRIA1 spans over 320 kb, and it is composed of 16 small exons transcribed in a 3242 bp mRNA (NCBI database; ref. seq. NM 00827).

Like all the other members of the AMPA receptor family, GluR1 occurs in two isoforms (flip and flop), which are generated by alternative splicing and are developmentally regulated [36]. Recent evidence has emerged indicating that glutamatergic mechanisms also exist in a variety of non-neural cells. Furthermore, it has recently been shown that glutamate has a role not only as a neurotransmitter but also as an immunomodulator. Glutamate interacts directly with T cell expressed glutamate receptors, leading to the activation or suppression of various T cell functions (cytokine secretion, proliferation, integrin-mediated adhesion, and migration). Asparaginase allergic reactions belong to type I hypersensitivity reactions or TH2 responses orchestrated by T helper 2 cells. Ca^2+^ signals are critical for T cell function. Several ion channels regulate Ca^2+^ influx from the extracellular space in T cells, either by conducting Ca^2+^ ions or by modulating the membrane potential that provides the driving force for Ca^2+^ influx [31,37].

Intronic polymorphisms in the glutamate receptor subunit GluR2 are the ones that direct RNA editing with consequential impacts on Ca^2+^ permeability and influx rate. All studied SNPs are in intronic sequences of the GRIA1 gene. The precise mechanism of intronic SNPs’ influence on allergy occurrence is not yet clear. RNA editing is a term for introducing sequence changes in gene transcripts. The editing of nuclear transcripts is catalyzed by deaminases (adenosine deaminases acting on RNA (ADARs)). The editing-created codon changes in GluRs affect amino acid positions with a critical impact on the properties of the glutamate-activated cation channels. This change, as a consequence, influences the gated AMPA channel’s permeability for Ca^2+^ ions. Thus, the possible mechanism of intronic SNPs’ influence on T-cell function and TH2 response development may be by Ca^2+^ influx changes [38,39].

The gene most frequently associated with the modulation of ASNase hypersensitivity reactions is GRIA1 [10]. This correlation has been substantiated across various childhood ALL studies, including those conducted by [32,40,41]. Specifically, the polymorphism rs4958351, situated on chromosome 5 (3807G>A), represents a transitional substitution, with its risk allele being A [32,40,41].

In one of the pioneering studies aiming to identify genetic polymorphisms that could contribute to the risk of allergy in a population of 485 children with ALL treated with ASNase, they analyzed more than 500,000 SNVs in a genome-wide association study [31]. They found that rs4958351 and four additional intronic SNVs of the GRIA1 gene were associated with ASNase hypersensitivity, rs10070447, rs6890057, rs4958676, and rs6889909.

Regarding the rs495835 single-nucleotide variant (SNV), a correlation was observed between the number of copies of the A allele and the risk of allergy to ASNase in patients across low-risk and standard/high-risk treatment arms. The cumulative incidence of ASNase allergy was notably different among patients with various genotypes, at 74%, 44%, and 32% in patients with the AA, AG, and GG genotypes, respectively. Additionally, the study revealed a significant association between allergy frequency and racial ancestry, with White children exhibiting a higher frequency of reactions compared with Black or Hispanic patients. Furthermore, the risk of allergy was found to be more pronounced in patients receiving *E. coli* ASNase compared with those receiving PEG-ASNase [40].

The remaining polymorphisms are specifically situated with identifiable risk alleles as follows: rs10070447 (2386-3102C>T) with its risk allele being T; rs6890057 (1824-20506C>T) with its corresponding risk allele being T; rs4958676 (2270+2302G>A) with its risk allele being A; and rs6889909 (1824-20561C>T) with its risk allele being T. These details have been corroborated by Chen (2010) and the National Center for Biotechnology Information’s SNP database (https://www.ncbi.nlm.nih.gov/snp) (accessed on 7 March 2024) [40].

Further investigations by Rajic et al. (2015) demonstrated significant associations between several single-nucleotide variants (SNVs)—namely, rs4958351, rs4958676, rs6889909, rs6890057, and rs10070447—within the Caucasian population and the incidence of hypersensitivity reactions to ASNase, particularly in those categorized as high-risk. Specifically, for rs4958351, the A allele was identified as a risk allele across the entire cohort of ALL patients. Furthermore, the severity of allergic reactions showed a distinct association with specific genotypes: individuals with the rs4958351 GA or rs10070447 CT genotype tended to exhibit allergy grade 2, whereas those harboring the rs4958676, rs6889909, and rs6890057 GG or CC genotype were prone to allergy grades 2 and 3. Although the precise mechanism underlying the influence of these SNVs on hypersensitivity remains unclear, the findings lend support to the notion that variations in the GRIA1 gene contribute to ASNase hypersensitivity, potentially dictating clinical manifestation and severity [41].

Meanwhile, Kutszeki et al. (2015) found varying impacts of GRIA1 polymorphisms on *E. coli*-ASNase hypersensitivity across ALL subtypes, highlighting the need for subtype-specific analyses. Surprisingly, they initially observed that the rs4958351 SNV did not exhibit any significant association with hypersensitivity within their entire study cohort. However, a notable divergence emerged after dissecting the data by ALL subtypes, namely, T-cell and pre-B-cell ALL. Specifically, they identified a significant difference in the association between the rs4958351 SNV and *E. coli*-ASNase hypersensitivity across these subgroups. Notably, the impact of the A allele on susceptibility to ASNase hypersensitivity dramatically varied between the different ALL subtypes. Among patients categorized under the T-ALL subgroup, those harboring the rs4958351 AA/AG genotype displayed a significantly reduced risk of developing hypersensitivity reactions compared with those with the GG genotype. Conversely, in the pre-B-cell ALL subgroup, while not statistically significant, a trend toward an increased risk of high-grade hypersensitivity was observed among individuals with the AA/AG genotype. These nuanced findings underscore the complex interplay between genetic variations within the GRIA1 gene and the manifestation of hypersensitivity reactions to ASNase, emphasizing the importance of subtype-specific analyses in pharmacogenetic studies [32].

A study conducted by Tanaka et al. (2016) aimed to investigate the potential link between ASNase hypersensitivity and genetic variations within the GRIA1 gene among 472 Japanese childhood ALL patients. Surprisingly, the SNV rs4958351 in GRIA1 did not exhibit any significant association with ASNase hypersensitivity (*p* = 0.99). This absence of correlation contrasts with findings from studies involving populations of European descent, suggesting potential ethnic-specific disparities in the genetic makeup surrounding these variants. For instance, notable differences in the minor allele frequency of the rs4958351 SNP were observed between Japanese (0.01) and European (0.36) populations, highlighting the influence of ethnic diversity on genetic susceptibility. Furthermore, discrepancies in ASNase type and therapeutic protocols could further contribute to the inconsistencies observed when compared with previous reports [33].

In summary, GRIA1 emerges as a key player in modulating hypersensitivity reactions to ASNase, with its genetic variations influencing the clinical manifestation and severity of such reactions. Nonetheless, further research is warranted to elucidate the underlying mechanisms and ethnic-specific disparities observed in different populations.

### 3.2. NFATC2

Another intriguing gene of interest is the nuclear factor of activated T cells 2 (NFATC2), which codes for a cytoplasmic component of the NFAT transcription factor family. Upon stimulation of the T-cell receptor, cytoplasmic NFATC2 undergoes dephosphorylation and translocation to the nucleus, where it plays a crucial role in gene regulation. While the specific impact of NFATC2 on the risk of drug-induced allergies remains unclear, several studies suggest its involvement in influencing the development and function of regulatory T cells, thereby modulating the immune response. Research utilizing NFAT inhibitors in various immune-related disease models has demonstrated that inhibiting the NFAT pathway can mitigate immune responses [10,42].

The single-nucleotide variant (SNV) rs6021191 resides within an intronic region on chromosome 20q13.2 and is situated in a genomic locus exhibiting weak enhancer activity in HUVEC and K562 cell lines. This suggests a potential role for the SNV in modulating the expression of NFATC2, with its risk allele identified as T. A pioneering investigation linking this gene to drug-induced hypersensitivity was conducted by Fernandez et al., who revealed an association between the variant and the susceptibility to ASNase hypersensitivity, further noting a correlation with elevated NFATC2 messenger RNA expression levels [43].

In a study mentioned above that sought to explore the relationship between ASNase hypersensitivity and genetic variations in a cohort of Japanese children, no significant association was found between NFATC2 rs6021191 and ASNase hypersensitivity (*p* = 0.76). These findings underscore the complexity of genetic influences on hypersensitivity reactions, highlighting the potential contribution of varying ASNase types and therapeutic protocols to the observed inconsistencies in previous reports [33].

### 3.3. CNTO3

CNOT3 is an integral component of the CCR4-not complex, known for its regulatory influence on gene expression and cellular signaling pathways. Studies have demonstrated that downregulation of CNOT3 leads to increased transcription of major histocompatibility complex (MHC) class II molecules, thereby influencing the regulation of HLA genes. However, despite its role in gene regulation, CNOT3 has also been identified as a tumor suppressor frequently mutated in T-cell acute lymphoblastic leukemia (T-ALL) [42,44].

In a recent study by Hojfeldt et al., the genetic predisposition to hypersensitivity reactions to PEG ASNase was investigated by defining the phenotype based on clinical hypersensitivity and lack of enzyme activity. This pioneering study, the first of its kind, incorporated measurements of enzyme activity to identify ASNase hypersensitivity. The genome-wide association study (GWA) analyzed 59 cases and 772 controls, revealing a significant association between an SNV (rs73062673) in an intronic region on chromosome 19q13.42 and PEG-ASNase allergy. Specifically, the risk allele (C) showed a strong correlation with PEG-ASNase allergy (*p* = 4.68 × 10^−8^), with the frequency of PEG-ASNase allergy varying among different genotypes (5% TT, 14.1% TC, and 41.7% CC). Interestingly, when silent inactivation patients were used as cases in the GWAS, the identified hypersensitivity SNVs were not associated with silent inactivation, suggesting distinct pathophysiological mechanisms underlying the two phenotypes [45].

### 3.4. ARHGAP28

Rho family is a member of the Ras supergene family of guanosine triphosphatase (GTPase), which is involved in cell morphology, gene transcription, the cell cycle, cell carcinogenesis, and other processes. However, the Rho GTPase-activating proteins (RHOGAPs) family is a negative regulatory factor of the Rho family proteins. ARHGAP proteins are altered in expression in many diseases, including cancer, so they may be a potential target for treating diseases. However, the deep regulatory mechanism of ARHGAP on the Rho family still needs to be clarified. ARHGAP28 was identified as a GTPase-activating protein (GAP) of a low-molecular-weight G-protein, the RhoA protein. It has been reported that overexpression of ARHGAP28 can inhibit the proliferation, migration, and invasion of osteosarcoma cells [46].

In 2021, Liu et al. identified ARHGAP28 rs9958628 as the top genetic variant associated with PEG-ASNase hypersensitivity in non-European ancestry with genome-wide significance. Variants in the ARHGAP28 locus were previously associated with poor responses to corticosteroid treatment in asthma patients, underscoring the immune regulatory function of this locus and the complex genetic interplay underlying hypersensitivity reactions. The rs9958628 located in the 5′-UTR of the Rho GTPase-activating protein 28 (ARHGAP28) gene transcript, in chromosome 18, was the top genetic variant and reached genome-wide significance in its association with hypersensitivity (*p* = 8.9 × 10^−9^; OR: 3.69) [47].

A study by Shastri et al. showed a combination of three SNPs (in ARHGAP28, CNOT3, and NFATC2) associated with PEG hypersensitivity in a cohort of 126 pediatric patients. Instead of a single SNP association approach, identifying combinations of variations in pathway-specific genes provides a more robust means to predict drug responses [48].

### 3.5. MYBBP1A

MYBBP1A, a ubiquitous 160 kDa protein, initially garnered attention for its role in binding and repressing the proto-oncogene c-MYB. Subsequent research has unveiled its multifaceted functions as a co-repressor of various transcription factors, positioning it as a tumor suppressor pivotal in regulating cellular evolution and malignancy. MYBBP1A plays a crucial role in diverse cellular processes, including mitosis, cell cycle control, the response to nuclear stress, ribosomal DNA synthesis, and tumoral suppression through the modulation of p53 activity [42,49]. Located on chromosome 17 in the 17p13.2 region, the MYBBP1A gene exhibits frequent loss of heterozygosity (50–80%) across different cancer types, such as sporadic breast and ovarian cancer, medulloblastomas, astrocytomas, osteosarcomas, leukemias, bladder cancer, and lung cancer. This loss of heterozygosity suggests the presence of one or more tumor suppressor genes within this genomic region [50].

In a study carried out in 2017, an association between the MYBBP1A gene and the development of allergy related to ASNase was made. The study particularly highlighted the SNP rs3809849 (G>C) as significant (*p* = 0.0006). The pivotal research marked the first demonstration of a link between the MYBBP1A gene and allergy risk [39].

### 3.6. HLA

The human leukocyte antigen (HLA), also referred to as the major histocompatibility complex (MHC) in humans, spans a genomic region of approximately 3.6 Mb situated in band 6p21.3 of the short arm of chromosome 6. This complex plays a pivotal role in both the innate and adaptive arms of the immune system, making it a cornerstone of immune function. Within this region lies a multitude of highly polymorphic genes characterized by strong linkage disequilibrium, reflecting the intricate genetic diversity within the human population. Given its significance in immunity and its implication in numerous autoimmune disorders, extensive efforts have been dedicated to the sequencing, analysis, and annotation of the MHC region [51].

Incorporating genes from the human leukocyte antigen (HLA) classes I, II, and III, the MHC region plays a pivotal role in immune surveillance and response mechanisms. Of notable significance within this region are the class II HLA molecules, such as HLA-DP, -DQ, and -DR, prominently displayed on the surfaces of antigen-presenting cells. These molecules serve as vital mediators, fostering communication between the innate and adaptive arms of the immune system and potentially contributing to allergic reactions and other immune-related phenomena [52].

In 2014, Fernandez et al. conducted a study to explore the associations of HLA class II alleles with hypersensitivity reactions to ASNase. Their investigation encompassed 1870 patients of European ancestry, employing molecular methods for HLA testing. The DNA sequencing targeted HLA-DRB1 and HLA-DQB1, focusing on exon 2 amplification. The researchers reported a robust correlation between the HLA-DRB107:01 allele and hypersensitivity in both cohorts. They demonstrated that HLA-DRB1 alleles conferring high-affinity binding to ASNase epitopes contributed to a heightened frequency of hypersensitivity. In their discovery cohort, they identified a significant association between HLA-DRB107:01 and ASNase hypersensitivity (*p* = 0.001), which was replicated in the validation cohort (*p* = 0.014). Additionally, they found polymorphic amino acid positions in HLA-DRB1 associated with ASNase hypersensitivity, with amino acid position 73, particularly glycine (Gly) residue carriers, showing a higher incidence of hypersensitivity compared with alanine (Ala/Ala) carriers [18].

Subsequently, in 2015, Fernandez et al. further substantiated the significance of the HLA-DRB107:01 allele in ASNase hypersensitivity. Their focus shifted to nonsynonymous SNPs, with the minor allele at rs17885382 in the HLA-DRB1 gene demonstrating the strongest association with ASNase hypersensitivity. This variant, located in exon 2 of the HLA-DRB1 gene (Arg.Gln,54), was previously implicated in their candidate gene analysis of HLA alleles associated with ASNase allergies. Notably, HLA-DRB107:01 emerged as the sole allele among those encoding Gln at position 54 with a minor allele frequency (MAF) > 1% [43].

In a study by Kutszeki et al. in 2017, the typing of HLADRB1, HLADQB1, and HLADQA1 alleles in 359 Hungarian pediatric ALL patients was conducted using NGS methods. The sequencing targeted exon 2 in HLA-DRB1 and exons 2 and 3 in HLA-DQB1 genes. Patients carrying HLA-DRB107:01 and HLA-DQB102:02 alleles exhibited a significantly higher risk of developing ASNase hypersensitivity compared with non-carriers. Furthermore, the HLA-DRB107:01–HLA-DQA102:01–HLA-DQB102:02 haplotype was significantly associated with an increased risk. Additionally, significant associations were found at 27 amino acid positions in HLA class II alleles, with a valine at position 78 in HLA-DRB1 demonstrating the strongest association with hypersensitivity. Interestingly, there was a notable difference in the proportion of HLA-DQB102:02 carriers between patients with pre-B-cell and T-cell ALL, suggesting potential subtype-specific susceptibility [32].

A genome-wide association study (GWAS) identified signals on chromosome 6 in an HLA-associated region, with the HLA-DQA1 variant (rs9272131) showing a significant association with ASNase hypersensitivity. This variant was located on 6p21.32 and was in high linkage disequilibrium with a cluster of SNVs located between the HLA-DQA2, HLA-DQA1, and HLA-DQB1 genes. The cluster of SNVs was shown to significantly increase the expression of HLA-DQA2, suggesting a plausible link between increased expression of this gene and the risk of hypersensitivity reactions. This finding underscores the pivotal role of the HLA region, particularly the HLA-DQ genes, in the antigen-presenting process and mediation of humoral immunity. Several large GWASs on different types of allergic reactions have highlighted the importance of SNVs in this specific region, emphasizing their essential role in allergic reactions [45].

Gagné et al. further corroborated the role of HLA alleles in ASNase-related hypersensitivity in childhood ALL in 2020. They identified a combination of HLA alleles—DRB107:01, DQA102:01, and DQB102:02—as potential markers for hypersensitivity. The combination of HLA alleles DRB107:01, DQA102:01, and DQB102:02 was associated with a higher risk of ASNase hypersensitivity. They concluded that HLA alleles serve as potential markers for ASNase-related hypersensitivity in childhood ALL, with the presence of DQB1*02:02 primarily contributing to the elevated risk [53].

In a study by Kutszeki et al. in 2020, 241 patients with a previously determined HLA-DRB107:01–DQA102:01–DQB102:02 haplotype and known ASNase hypersensitivity status were evaluated. The researchers identified two SNPs, rs28383172 and rs7775228, as the most suitable tags for the HLA-DRB107:01–DQA102:01–DQB102:02 haplotype. The role of rs28383172 as a cross-ethnicity tagging SNP for HLA-DRB107:01 carrier status was previously predicted. Additionally, rs28724121 and rs17885382 SNPs were also used to tag the HLA DRB107:01 allele. The researchers confirmed that rs28383172 is an ideal and sufficient surrogate marker for HLA-DRB1*07:01 carrier status [54].

Expanding on these findings, a study by Liu et al. in 2021 conducted the first HLA allele and GWAS to identify loci associated with hypersensitivity reactions to PEG-ASNase in diverse cohorts of pediatric patients. They found that HLA alleles DQB102:02, DRB107:01, and DQA102:01 exhibited the strongest associations with PEG-ASNase hypersensitivity, particularly in patients of European ancestry. The top allele HLA-DQB102:02 was tagged by HLA-DQB1 rs1694129, associated with PEG-ASNase hypersensitivity reactions in European ancestry and partially replicated in non-European ancestry. Notably, all SNPs associated with PEG-ASNase hypersensitivity reaching genome-wide significance in European ancestry were in class II HLA loci and were partially replicated in non-European ancestry. Interestingly, the presence of HLA-DRB107:01 alone increased the risk of reactions only when accompanied by DQB102:02. Patients harboring the full haplotype with all three HLA-DRB107:01, -DQA102:01, and -DQB102:02 alleles tended to be at higher risk for reactions than those with only HLA-DRB107:01 and -DQA102:01 alleles but not -DQB102:02 [55].

In a study by Kondily et al. in 2021, it was demonstrated that the increased risk of hypersensitivity associated with HLA-DRB107:01 was present only in the *E. coli* ASNase group, while a higher frequency of the DRB107:01 and DQA1*02:01 combination was similar for both ASNase preparations when patients with and without allergies were compared [56].

The study of associations between HLA alleles and hypersensitivity reactions to ASNase has provided valuable insights into the immunogenetic mechanisms underlying these adverse responses. While some studies have shown consistent associations between specific HLA alleles and hypersensitivity reactions, others still report conflicting results, highlighting the complexity and ongoing need for research in this field. A comprehensive understanding of these genetic-immunological interactions may guide future, more personalized, and effective therapeutic approaches in managing adverse reactions to ASNase.

Table 2 presents an association of recent studies relating pharmacogenetics and ASNase.

## 4. Conclusions

The rapid advancements in pharmacogenetics, marked by increasingly accessible genotyping and sequencing services with reduced costs and turnaround times, are driving significant growth in this field. These technological strides enable deeper exploration through complex analyses, expanding the catalog of validated genetic markers capable of predicting treatment outcomes and risks. Consequently, there is a shifting paradigm from traditional trial-and-error dosing toward personalized pharmacogenetic approaches tailored to individual genetic profiles.

This comprehensive review of published data underscores the emerging evidence implicating specific genes in ASNase hypersensitivity. Numerous studies have highlighted significant associations between single-nucleotide variants (SNVs) within these genes and hypersensitivity reactions. Notably, most pharmacogenetic investigations of hypersensitivity have focused on patients with acute lymphoblastic leukemia (ALL) treated with ASNase derived from *E. coli*, emphasizing the need for broader exploration across different formulations. Future research investigating these variants holds promise for advancing our understanding of ASNase pharmacogenetics. By uncovering population-specific patterns, such studies seek to optimize treatment strategies and enhance patient outcomes through tailored therapeutic interventions.

## Figures and Tables

**Table 1:** Pharmacokinetic data.

Types of ASNase	Derived From	Half-Life	Asparagine Depletion
*E. coli* native	*Escherichia coli*	1.3 days	14–23 days
Erwinase	*Erwinia carotovora*	0.65 day	7–15 days
PEG-ASNase	*E. coli* with polyethylene glycol	7 days	26–34 days

**Table 2:** Summary of key findings from pharmacogenetic studies of asparaginase, highlighting genetic associations and relevant findings in the literature.

Author	Gene	Variant	Allele	ASNase	Method	Population	*p*-Value	Conclusions
Chen et al., 2010 [31]	GRIA1	rs4958351 rs10070447 rs6890057 rs4958676 e rs6889909	G>A C>T C>T G>A C>T	*E. coli*	GWAS	485 American children: 322 in a discovery cohort and 163 in a validation cohort	3.5 × 10^−7^	Genetic variations were associated with risk of allergy to ASNase.
Rajic et al., 2015 [41]	GRIA1	rs4958351 rs4958676 rs6889909 rs6890057 rs10070447	G>A G>A C>T C>T C>T	*E. coli* and PEG ASNase	Gene candidate	146 Slovenian patients	*p* < 0.05	rs4958351 or rs10070447 are associated with allergy grade 2, while rs495867, rs6889909, and rs6890057 are associated with allergy grades 2 and 3.
Kutszeki, et al., 2015 [32]	GRIA1	rs4958351	G>A	*E. coli*	Gene candidate	576 Hungarian pediatric	pre-B ALL *p* > 0.05 T-ALL *p* < 0.05	Genetic variants might influence the risk of ASNase hypersensitivity, but subgroups of patients can significantly differ.
Tanaka, et al., 2016 [33]	GRIA1 NFACT2	rs4958351 rs6021191	G>A A>T	*E. coli*	GWAS	472 Japanese children	*p* > 0.05	They did not find an association.
Fernandez et al., 2014 [18]	HLA-DR	HLA-DRB1*07:01		*E. coli*	Gene candidate	1870 patients of European ancestry: 541 in discovery cohort and 1329 in validation cohort	7.5 × 10 ^−5^	Higher incidence of hypersensitivity and anti-ASNase antibodies in patients with HLA-DRB1*07:01 alleles.
Fernandez et al., 2015 [43]	NFACT2 HLA-DRB1	rs6021191 rs17885382	A>T C>T	*E. coli* and PEG ASNase	GWAS	*n* = 3308 patients of diverse ancestry	4.1 × 10^−8^ 3.23 × 10^−26^	rs6021191 was associated with an increased risk of hypersensitivity, and they confirmed the importance of the HLA-DRB1*07:01 allele.
Kutszeki, et al., 2017 [32]	HLA-DQA1 HLA-DQB1	HLA-DQA1*02:01 HLA-DQB1*02:02		*E. coli*	GWAS	*n* = 359 Hungarian pediatric ALL	4.56 × 10^−5^ *p* = 4.56 × 10^−5^	HLA-DRB1*07:01–HLA-DQA1*02:01–HLA-DQB1*02:02 haplotype was associated with a high risk of *E. coli* hypersensitivity.
Gagné et al., 2020 [53]	HLA-DRB1 HLA-DQA1 HLA-DQB1	DRB1*07:01 DQA1*02:01 DQB1*02:02		*E. coli*	Gene candidate	French/Canadian children: 284 in discovery cohort and 243 in validation cohort	*p* = 0.006	Combination of HLA alleles, DRB1*07:01, DQA1*02:01, and DQB1*02:02 was associated with a higher risk of ASNase hypersensitivity.
Kutszeki et al., 2020 [54]	HLA-DRB1*07:01 HLA-DQ2.2	rs28383172 rs7775228	A>G T>C	*E. coli*	Gene candidate	241 patients	*p* < 0.01	HLA-DRB1*07:01–DQA1*02:01–DQB1*02:02 haplotype was associated with *E. coli* ASNase hypersensitivity and a combination of 2 SNPs—rs28383172 and rs7775228—as a tag for this haplotype.
Liu, et al., 2021 [55]	HLA-DQB1 ARHGAP28	rs1694129 rs9958628	G>T A>T	PEG ASNase	GWAS	Three cohorts: *n* = 598, B- and T-lineage ALL; *n* = 2472, B-ALL; *n* = 1189, T-ALL	*p* = 1.1 × 10^−8^ *p* = 8.9 × 10^−9^	The HLA-DRB1*07:01-DQA1*02:01-DQB1*02:02 haplotype was associated with hypersensitivity to PEG-ASNase. Also identified ARHGAP28 rs9958628 as associated with PEG-ASNase hypersensitivity in non-European ancestry.
Kondill et al., 2021 [56]	HLA-DRB1*07:01 HLA-DQB1*02:02	rs28724121 rs281863414		PEG ASNase	Gene candidate	199 French–Canadian patients	*p* = 0.04	HLA-DRB1*07:01- DQA1*02:01 haplotype was associated with hypersensitivity to PEG-ASNase in patients.
Hojfeldt, et al., 2019 [45]	CNTO3 HLA-DQA1 TAP2	rs73062673 rs9272131 rs115360810	C/T C>T A>G	PEG ASNase	GWAS	Nordic patients: 59 cases and 772 controls	4.68 × 10^−8^ 9.37 × 10^−6^	They associated hypersensitivity with CNOT3, relating to a possible involvement of an HLA-regulating gene. Also identified a genetic variant on chromosome 6 in close relation to HLA genes.
Shastri et al., 2023 [48]	*CNTO3* *ARGHAP28* *NFACT2*	rs73062673 rs9958628 rs6021191	C>T A>T A>T	PEG ASNase	Gene candidate	126 patients		They showed a combination of 3 SNPs associated with PEG hypersensitivity. Instead of an SNP association approach, identifying combinations of variations in pathway-specific genes provides a more robust means to predict drug responses.
Abaji et al., 2017 [39]	*MYBBP1A*	rs3809849	G>C	*E. coli*	WES	North American children: discovery cohort = 302; replication cohort = 282	*p* = 6 × 10^−4^	This variant was associated with allergy.

Genome-wide association study (GWAS); whole-exome sequencing (WES).
